# Supplementary material for: The interactome of intact mitochondria by cross-linking mass spectrometry provides evidence for coexisting respiratory supercomplexes
Source: Mol Cell Proteomics. 2017 Dec 8;17(2):216–32. doi: 10.1074/mcp.RA117.000470 (PMC5795388; doi:10.1074/mcp.RA117.000470)
Supplement: Supplemental Data [file supp_RA117.000470_133922_0_supp_23978_fzffwf.zip › spectra_annotation/mito_spectra_annotation/426-1-4-1-5-1.pdf]

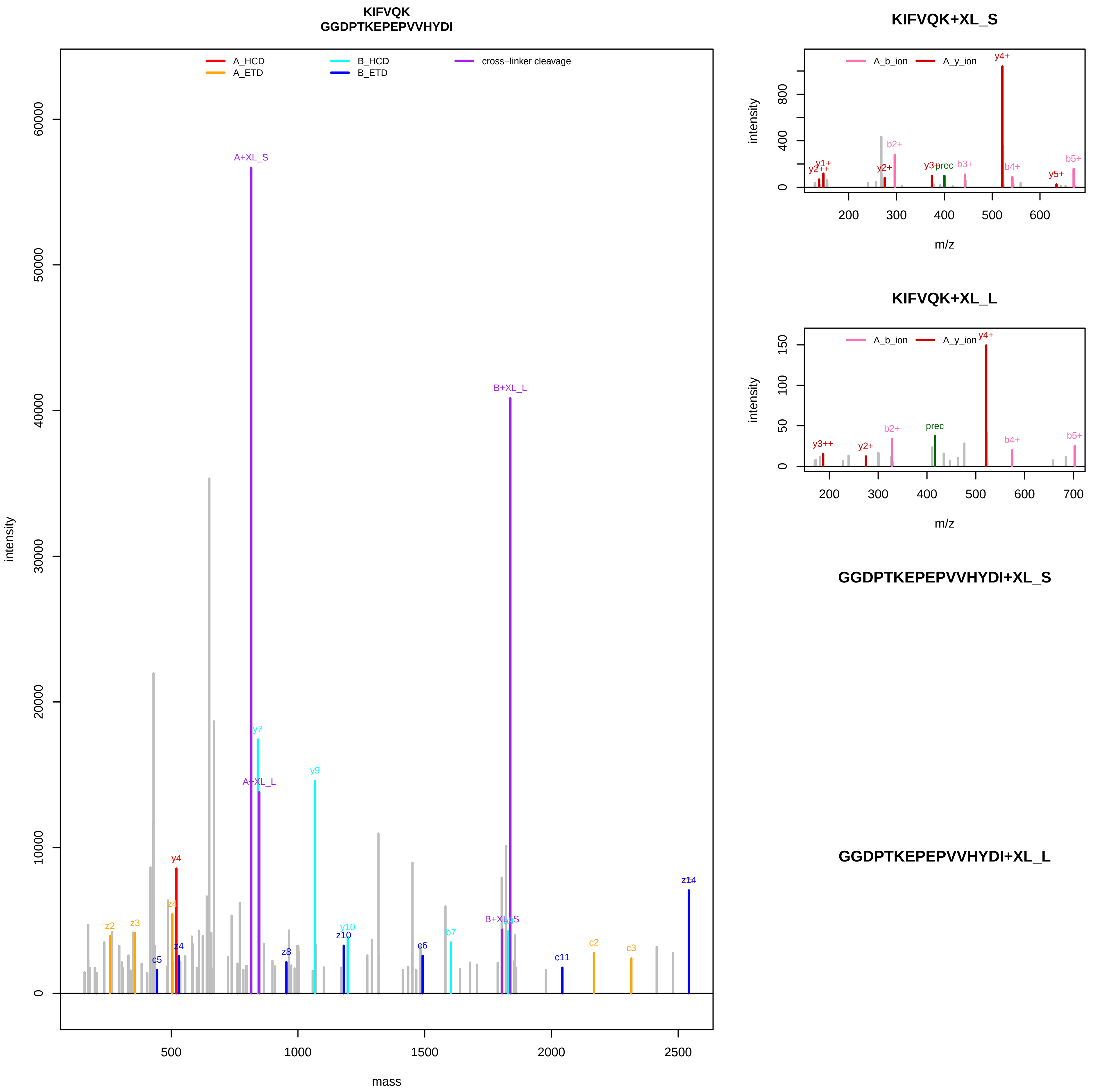

KIFVQK
GGDPTKEPEPVVHYDI
A_HCD
A_ETD
B_HCD
B_ETD
cross−linker cleavage
intensity
mass
A+XL_S
B+XL_L
A+XL_L
B+XL_S
y4
y7
y9
y10
b7
z2
z3
z4
c5
z4
z8
z10
c6
c11
c2
c3
z14
KIFVQK+XL_S
A_b_ion
A_y_ion
y1+
y2++
y2+
b2+
y3+
prec
b3+
y4+
b4+
y5+
b5+
m/z
KIFVQK+XL_L
y3++
GGDPTKEPEPVVHYDI+XL_S
GGDPTKEPEPVVHYDI+XL_L
